# Bis(tetra­phenyl­phospho­nium) tetra­sulfido­tungstate(VI)

**DOI:** 10.1107/S1600536808007472

**Published:** 2008-03-29

**Authors:** Patricia Leyva Bailen, Anthony V. Powell, Paz Vaqueiro

**Affiliations:** a Department of Chemistry, Heriot–Watt University, Edinburgh EH14 4AS, Scotland

## Abstract

The crystal structure of the title compound, (C_24_H_20_P)_2_[WS_4_], which was prepared under hydro­thermal conditions, contains tetra­phenyl­phospho­nium cations linked by supra­molecular inter­actions into chains running along the [110] and [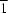
10] directions. The [WS_4_]^2−^anions, which lie on twofold axes, are located in the cavities created between the cation chains.

## Related literature

Isostructural compounds include [Ph_4_P]_2_[MoSe_4_] and [Ph_4_P]_2_[WSe_4_] (O’Neal & Kolis, 1988 ▶), [Ph_4_P]_2_[NiCl_4_] (Ruhlandt-Senge & Müller, 1990 ▶), and [Ph_4_P]_2_[CdBr_4_] and [Ph_4_P]_2_[HgBr_4_] (Hasselgren *et al.*, 1997 ▶). The related compounds [NH_4_]_2_[WS_4_] and [Ph_4_P][W(HS)S_3_] were reported by Sasvári (1963 ▶) and Parvez *et al.* (1997 ▶), respectively. For a review on thio­metalates, see Müller *et al.* (1981 ▶). Supra­molecular inter­actions between tetra­phenyl­phospho­nium cations have been discussed by Dance & Scudder (1995 ▶, 1996 ▶). 
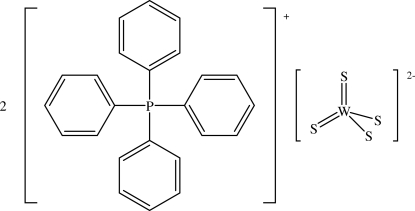


## Experimental

### 

#### Crystal data

(C_24_H_20_P)_2_[WS_4_]
*M* *_r_* = 990.86
Monoclinic, 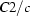

*a* = 11.1069 (4) Å
*b* = 19.4557 (6) Å
*c* = 20.2373 (6) Å
β = 91.242 (2)°
*V* = 4372.1 (2) Å^3^
*Z* = 4
Mo *K*α radiation
μ = 2.94 mm^−1^
*T* = 293 K
0.40 × 0.30 × 0.30 mm

#### Data collection

Bruker–Nonius APEXII CCD area-detector diffractometer
Absorption correction: multi-scan (*SADABS*; Sheldrick, 1996 ▶) *T*_min_ = 0.351, *T*_max_ = 0.414
26702 measured reflections
6646 independent reflections
4008 reflections with *I* > 3σ(*I*)
*R*_int_ = 0.044

#### Refinement

*R*[*F* ^2^ > 2σ(*F* ^2^)] = 0.027
*wR*(*F* ^2^) = 0.031
*S* = 1.11
4008 reflections
249 parameters
H-atom parameters constrained
Δρ_max_ = 1.47 e Å^−3^
Δρ_min_ = −0.63 e Å^−3^

### 

Data collection: *APEX2* (Bruker, 2005 ▶); cell refinement: *APEX2*; data reduction: *APEX2*; program(s) used to solve structure: *SIR92* (Altomare *et al.*, 1994 ▶); program(s) used to refine structure: *CRYSTALS* (Betteridge *et al.*, 2003 ▶); molecular graphics: *ATOMS* (Dowty, 2000 ▶); software used to prepare material for publication: *CRYSTALS*.

## Supplementary Material

Crystal structure: contains datablocks I, global. DOI: 10.1107/S1600536808007472/cs2070sup1.cif

Structure factors: contains datablocks I. DOI: 10.1107/S1600536808007472/cs2070Isup2.hkl

Additional supplementary materials:  crystallographic information; 3D view; checkCIF report

## Figures and Tables

**Table:** 

W1—S2^i^	2.1962 (10)
W1—S3^i^	2.1915 (9)
W1—S2	2.1962 (10)
W1—S3	2.1915 (9)

**Table:** 

S2^i^—W1—S2	110.82 (7)
S3^i^—W1—S2	108.59 (4)
S3^i^—W1—S3	110.02 (6)
S2—W1—S3	109.40 (4)

Symmetry code: (i) 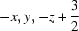.

**Fig. 1.**
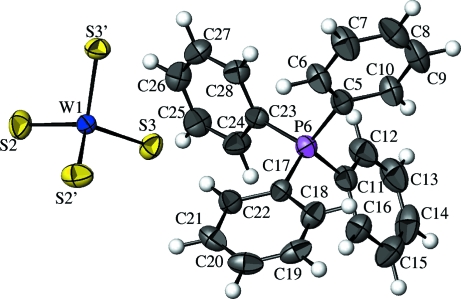
Local coordination diagram for [Ph4P]2[WS4] showing the atom labelling scheme and displacement ellipsoids at 50% probability for non-H atoms. H atoms are shown as circles of arbitrary radii. [Symmetry code: (i) -x, y, 3/2 - z]

**Fig. 2.**
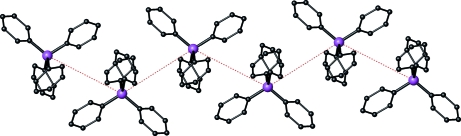
Zigzag chain of [Ph4P]+cations, running along the b axis, illustrating the supramolecular edge-to-face phenyl interactions. Short P···P distances (ca 6.5 Å) are shown as red lines.

**Fig. 3.**
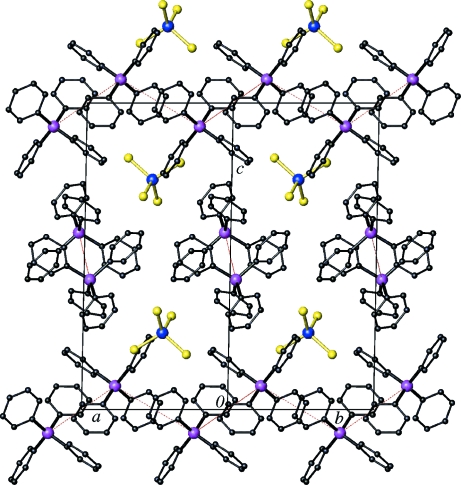
View of the [Ph4P]2[WS4] structure along the [110] direction. The relatively short P···.P distances within the zigzag chains are shown as red lines. H atoms have been omitted for clarity.

## supplementary crystallographic information

### Comment

The ability of tetrathiometallates to act as multidentate ligands has resulted in a rich coordination chemistry, which includes both discrete multimetal clusters such as [A(MS_4_)_2_]^2-^ (A = Fe, Co, Ni, Pd, Pt, Zn, Cd; *M* = Mo, W) and extended structures such as NH_4_Cu[WS_4_] (Müller *et al.*, 1981). The compound reported here is the result of our ongoing research efforts on the synthesis of novel transition metal thiometallates.

The title compound, which was prepared under hydrothermal conditions, contains discrete WS_4_^2-^ anions and tetraphenylphosphonium [Ph_4_P]^+^ cations. The local coordination and the atom-labelling scheme are shown in Figure 1. Each tungsten atom is surrounded by four sulfur atoms in a tetrahedral coordination. The S—W—S bond angles range from 108.59 (4) to 110.82 (7) whilst the four W—S distances are nearly identical ranging from 2.1915 (9) to 2.1962 (10) Å, and are similar to those found in [NH_4_]_2_[WS_4_] (Sasvári, 1963).

The [Ph_4_P]^+^ cations are arranged into zigzag chains (Figure 2) in which each phenyl group points towards another phenyl group in a neighbouring cation, with the H atoms of a given ring oriented towards the π electron density of the second phenyl ring. It has been proposed that attractive interactions between the phenyl groups of [Ph_4_P]^+^cations play a major role in the crystal packing of compounds of this type (Dance & Scudder, 1995). In particular, the so-called sextuple phenyl embrace, in which three phenyl groups of a [Ph_4_P]^+^ cation face three phenyl groups of an adjacent [Ph_4_P]^+^ cation in an edge-to-face conformation, is a frequently observed supramolecular motif (Dance & Scudder, 1996). As illustrated by Figure 2, the zigzag chains of [Ph_4_P]^+^cations found in the title compound could be described by considering each [Ph_4_P]^+^cation to interact with its two neighbours through sextuple phenyl embraces. The P···P distances within the chain are *ca* 6.5 Å, comparable to those observed in compounds containing this type of chain (Dance & Scudder, 1996). The structure of the title compound contains zigzag [Ph_4_P]^+^ chains running along the [110] and [110] directions (Figure 3). There are relatively short P···P distances, of *ca* 7.3 Å, between [Ph_4_P]^+^cations from different chains, which involve two face-to-face phenyl interactions, suggesting that there may be additional interchain supramolecular interactions. This type of interaction has been reported previously (Dance & Scudder,1996), and has been termed quadruple phenyl embrace. The tetrathiotungstate anions are located in the cavities created between the cation chains (Figure 3). A number [Ph_4_P]^+^ salts, containing chemically diverse tetrahedral anions, adopt a similar crystal structure. Isostructural compounds that have been reported include selenometallates such as [Ph_4_P]_2_[MoSe_4_] (O'Neal & Kolis, 1988) and [Ph_4_P]_2_[WSe_4_] (O'Neal & Kolis, 1988), and halometalates like [Ph_4_P]_2_[NiCl_4_] (Ruhlandt-Senge & Müller, 1990), [Ph_4_P]_2_[CdBr_4_] and [Ph_4_P]_2_[HgBr_4_] (Hasselgren *et al.*, 1997).

### Experimental

A mixture of [NH_4_]_2_[WS_4_] (0.348 g; 1 mmol) and [Ph_4_P]Br (0.21 g; 0.5 mmol) was loaded into a 23 ml Teflon-lined stainless autoclave, 2 ml of deionized water and 2 ml of ethylenediamine were added to the mixture. After stirring the mixture, the container was closed, heated at 443 K for 4 days, and cooled to room temperature at a cooling rate of 1 K min^-1^. The product was filtered, washed with deionized water, methanol and acetone and dried in air at room temperature. The product consists of large number of yellow crystals of the title compound (approximately 80% yield).

### Refinement

The H atoms were positioned geometrically, with *U*_iso_(H) = 1.2*U*_eq_(carbon).

### Crystal data

**Table:**

(C_24_H_20_P)_2_[WS_4_]	*F*_000_ = 1984
*M**_r_* = 990.86	*D*_x_ = 1.505 Mg m^−^^3^
Monoclinic, *C*2/*c*	Mo *K*α radiation λ = 0.71073 Å
*a* = 11.1069 (4) Å	Cell parameters from 6646 reflections
*b* = 19.4557 (6) Å	θ = 2.0–30.5º
*c* = 20.2373 (6) Å	µ = 2.94 mm^−^^1^
β = 91.242 (2)º	*T* = 293 K
*V* = 4372.1 (2) Å^3^	Block, yellow
*Z* = 4	0.40 × 0.30 × 0.30 mm

### Data collection

**Table:**

Bruker–Nonius APEXII CCD area-detector diffractometer	4008 reflections with *I* > 3σ(*I*)
Monochromator: graphite	*R*_int_ = 0.044
*T* = 293 K	θ_max_ = 30.5º
ω/2θ scans	θ_min_ = 2.0º
Absorption correction: multi-scan(SADABS; Sheldrick, 1996)	*h* = −15→15
*T*_min_ = 0.351, *T*_max_ = 0.414	*k* = −27→26
26702 measured reflections	*l* = −28→28
6646 independent reflections	

### Refinement

**Table:**

Refinement on *F*	Hydrogen site location: inferred from neighbouring sites
Least-squares matrix: full	H-atom parameters constrained
*R*[*F*^2^ > 2σ(*F*^2^)] = 0.027	Method, part 1, Chebychev polynomial, [*w*eight] = 1.0/[A_0_*T_0_(x) + A_1_*T_1_(x) ··· + A_n-1_]*T_n-1_(x)] where A_i_ are the Chebychev coefficients listed belo*w* and x = *F* /*F*max Method = Robust Weighting W = [*w*eight] * [1-(delta*F*/6*sigma*F*)^2^]^2^ A_i_ are: 2.09 -0.539 1.80
*wR*(*F*^2^) = 0.031	(Δ/σ)_max_ = 0.003
*S* = 1.11	Δρ_max_ = 1.47 e Å^−^^3^
4008 reflections	Δρ_min_ = −0.63 e Å^−^^3^
249 parameters	Extinction correction: None
Primary atom site location: structure-invariant direct methods	

### Fractional atomic coordinates and isotropic or equivalent isotropic displacement parameters (Å^2^)

**Table:**

	*x*	*y*	*z*	*U*_iso_*/*U*_eq_	
W1	0.0000	0.543792 (10)	0.7500	0.0336	
S2	0.10243 (10)	0.60787 (6)	0.82067 (6)	0.0600	
S3	0.12468 (8)	0.47920 (5)	0.69500 (5)	0.0484	
P4	0.40710 (8)	0.63367 (4)	0.57674 (4)	0.0388	
C5	0.3781 (3)	0.70067 (18)	0.63526 (17)	0.0437	
C6	0.3089 (5)	0.6844 (2)	0.6898 (2)	0.0563	
C7	0.2841 (6)	0.7352 (3)	0.7362 (2)	0.0707	
C8	0.3290 (5)	0.8004 (2)	0.7288 (2)	0.0686	
C9	0.3984 (5)	0.8169 (2)	0.6756 (3)	0.0689	
C10	0.4229 (4)	0.7669 (2)	0.6283 (2)	0.0573	
C11	0.5148 (4)	0.66260 (19)	0.5171 (2)	0.0487	
C12	0.4796 (5)	0.7113 (2)	0.4705 (2)	0.0628	
C13	0.5591 (6)	0.7353 (3)	0.4247 (3)	0.0736	
C14	0.6755 (6)	0.7104 (3)	0.4251 (2)	0.0784	
C15	0.7125 (4)	0.6610 (3)	0.4694 (3)	0.0736	
C16	0.6318 (4)	0.6367 (3)	0.5169 (2)	0.0587	
C17	0.4672 (3)	0.56172 (17)	0.62141 (17)	0.0385	
C18	0.5678 (4)	0.5713 (2)	0.6630 (2)	0.0510	
C19	0.6220 (4)	0.5167 (3)	0.6945 (2)	0.0565	
C20	0.5746 (4)	0.4515 (3)	0.68513 (19)	0.0595	
C21	0.4726 (4)	0.44106 (19)	0.6465 (2)	0.0550	
C22	0.4181 (3)	0.49626 (19)	0.61360 (18)	0.0445	
C23	0.2728 (3)	0.60850 (18)	0.53184 (17)	0.0396	
C24	0.2857 (4)	0.5749 (2)	0.4718 (2)	0.0528	
C25	0.1833 (4)	0.5528 (2)	0.43687 (19)	0.0583	
C26	0.0705 (4)	0.5641 (2)	0.4617 (2)	0.0538	
C27	0.0588 (4)	0.5965 (2)	0.5217 (2)	0.0542	
C28	0.1588 (3)	0.6183 (2)	0.55694 (18)	0.0471	
H61	0.2773	0.6368	0.6957	0.0679*	
H71	0.2337	0.7241	0.7750	0.0853*	
H81	0.3108	0.8363	0.7624	0.0823*	
H91	0.4312	0.8644	0.6708	0.0827*	
H101	0.4725	0.7788	0.5893	0.0687*	
H121	0.3955	0.7294	0.4702	0.0758*	
H131	0.5329	0.7706	0.3915	0.0887*	
H141	0.7338	0.7287	0.3926	0.0950*	
H151	0.7961	0.6423	0.4681	0.0883*	
H161	0.6583	0.6014	0.5500	0.0706*	
H181	0.6007	0.6186	0.6700	0.0612*	
H191	0.6946	0.5237	0.7237	0.0677*	
H201	0.6152	0.4114	0.7068	0.0719*	
H211	0.4376	0.3940	0.6421	0.0663*	
H221	0.3450	0.4889	0.5848	0.0533*	
H241	0.3675	0.5666	0.4538	0.0636*	
H251	0.1918	0.5287	0.3936	0.0699*	
H261	−0.0027	0.5489	0.4362	0.0647*	
H271	−0.0231	0.6043	0.5398	0.0652*	
H281	0.1494	0.6412	0.6007	0.0565*	

### Atomic displacement parameters (Å^2^)

**Table:**

	*U*^11^	*U*^22^	*U*^33^	*U*^12^	*U*^13^	*U*^23^
W1	0.03259 (9)	0.03114 (9)	0.03738 (10)	0.0000	0.00442 (6)	0.0000
S2	0.0597 (6)	0.0606 (6)	0.0602 (6)	−0.0175 (5)	0.0126 (5)	−0.0253 (5)
S3	0.0416 (4)	0.0516 (5)	0.0522 (5)	0.0069 (3)	0.0016 (4)	−0.0151 (4)
P4	0.0428 (4)	0.0362 (4)	0.0375 (4)	−0.0004 (3)	0.0005 (3)	0.0010 (3)
C5	0.0527 (19)	0.0390 (17)	0.0391 (17)	−0.0021 (14)	−0.0030 (14)	−0.0012 (13)
C6	0.080 (3)	0.0410 (19)	0.048 (2)	−0.0067 (18)	0.0104 (19)	−0.0017 (16)
C7	0.106 (4)	0.059 (3)	0.047 (2)	−0.010 (3)	0.015 (2)	−0.0086 (19)
C8	0.104 (4)	0.048 (2)	0.054 (2)	0.010 (2)	−0.006 (2)	−0.0130 (19)
C9	0.097 (4)	0.039 (2)	0.070 (3)	−0.008 (2)	−0.011 (3)	−0.0022 (19)
C10	0.073 (3)	0.043 (2)	0.056 (2)	−0.0090 (18)	0.0019 (19)	−0.0008 (17)
C11	0.056 (2)	0.0402 (17)	0.050 (2)	−0.0044 (15)	0.0076 (16)	−0.0003 (15)
C12	0.082 (3)	0.049 (2)	0.058 (2)	0.004 (2)	0.016 (2)	0.0115 (19)
C13	0.107 (4)	0.051 (2)	0.064 (3)	−0.009 (3)	0.021 (3)	0.009 (2)
C14	0.094 (4)	0.086 (4)	0.057 (3)	−0.044 (3)	0.024 (3)	−0.005 (3)
C15	0.051 (2)	0.100 (4)	0.070 (3)	−0.016 (2)	0.011 (2)	−0.004 (3)
C16	0.052 (2)	0.069 (3)	0.055 (2)	−0.0096 (19)	0.0031 (18)	0.000 (2)
C17	0.0371 (15)	0.0405 (17)	0.0380 (15)	−0.0014 (12)	0.0021 (12)	0.0007 (12)
C18	0.0441 (18)	0.063 (2)	0.0460 (19)	−0.0070 (17)	−0.0037 (15)	0.0060 (17)
C19	0.0375 (17)	0.086 (3)	0.046 (2)	0.0089 (18)	−0.0010 (15)	0.0100 (19)
C20	0.064 (2)	0.070 (3)	0.0445 (18)	0.030 (2)	0.0064 (16)	0.014 (2)
C21	0.073 (3)	0.041 (2)	0.051 (2)	0.0101 (17)	0.0088 (18)	−0.0020 (15)
C22	0.0474 (18)	0.0428 (18)	0.0432 (18)	0.0004 (14)	−0.0007 (14)	0.0007 (14)
C23	0.0432 (17)	0.0389 (16)	0.0367 (16)	0.0049 (13)	−0.0013 (13)	0.0034 (13)
C24	0.0441 (19)	0.067 (2)	0.048 (2)	0.0043 (17)	0.0033 (15)	−0.0106 (18)
C25	0.065 (2)	0.068 (3)	0.0409 (18)	−0.002 (2)	−0.0046 (16)	−0.0115 (18)
C26	0.057 (2)	0.061 (2)	0.0439 (19)	−0.0073 (17)	−0.0063 (16)	0.0061 (16)
C27	0.0419 (18)	0.067 (2)	0.054 (2)	−0.0001 (17)	0.0058 (16)	0.0019 (18)
C28	0.0487 (19)	0.054 (2)	0.0388 (17)	0.0032 (16)	0.0029 (14)	−0.0019 (15)

### Geometric parameters (Å, °)

**Table:**

W1—S2^i^	2.1962 (10)	C14—H141	0.999
W1—S3^i^	2.1915 (9)	C15—C16	1.409 (6)
W1—S2	2.1962 (10)	C15—H151	0.998
W1—S3	2.1915 (9)	C16—H161	0.999
P4—C5	1.795 (4)	C17—C18	1.396 (5)
P4—C11	1.807 (4)	C17—C22	1.393 (5)
P4—C17	1.788 (3)	C18—C19	1.371 (6)
P4—C23	1.797 (4)	C18—H181	0.999
C5—C6	1.395 (5)	C19—C20	1.384 (7)
C5—C10	1.390 (5)	C19—H191	0.999
C6—C7	1.394 (6)	C20—C21	1.378 (7)
C6—H61	0.998	C20—H201	0.998
C7—C8	1.374 (7)	C21—C22	1.395 (6)
C7—H71	0.997	C21—H211	0.999
C8—C9	1.376 (8)	C22—H221	0.999
C8—H81	0.998	C23—C24	1.390 (5)
C9—C10	1.394 (7)	C23—C28	1.388 (5)
C9—H91	0.999	C24—C25	1.393 (6)
C10—H101	0.999	C24—H241	0.999
C11—C12	1.388 (6)	C25—C26	1.378 (6)
C11—C16	1.394 (6)	C25—H251	1.000
C12—C13	1.375 (7)	C26—C27	1.377 (6)
C12—H121	0.999	C26—H261	0.998
C13—C14	1.380 (9)	C27—C28	1.374 (6)
C13—H131	0.999	C27—H271	0.999
C14—C15	1.371 (9)	C28—H281	0.999
			
S2^i^—W1—S3^i^	109.40 (4)	C14—C15—C16	119.6 (5)
S2^i^—W1—S2	110.82 (7)	C14—C15—H151	120.2
S3^i^—W1—S2	108.59 (4)	C16—C15—H151	120.2
S2^i^—W1—S3	108.59 (4)	C15—C16—C11	119.2 (4)
S3^i^—W1—S3	110.02 (6)	C15—C16—H161	120.4
S2—W1—S3	109.40 (4)	C11—C16—H161	120.4
C5—P4—C11	110.19 (17)	P4—C17—C18	119.1 (3)
C5—P4—C17	107.77 (16)	P4—C17—C22	121.2 (3)
C11—P4—C17	109.65 (17)	C18—C17—C22	119.7 (3)
C5—P4—C23	111.88 (17)	C17—C18—C19	121.0 (4)
C11—P4—C23	107.53 (18)	C17—C18—H181	119.6
C17—P4—C23	109.81 (16)	C19—C18—H181	119.5
P4—C5—C6	117.8 (3)	C18—C19—C20	119.0 (4)
P4—C5—C10	122.5 (3)	C18—C19—H191	120.6
C6—C5—C10	119.7 (4)	C20—C19—H191	120.4
C5—C6—C7	119.5 (4)	C19—C20—C21	121.2 (4)
C5—C6—H61	120.3	C19—C20—H201	119.3
C7—C6—H61	120.2	C21—C20—H201	119.5
C6—C7—C8	120.2 (4)	C20—C21—C22	120.0 (4)
C6—C7—H71	120.0	C20—C21—H211	120.0
C8—C7—H71	119.8	C22—C21—H211	120.0
C7—C8—C9	120.8 (4)	C21—C22—C17	119.0 (4)
C7—C8—H81	119.6	C21—C22—H221	120.4
C9—C8—H81	119.7	C17—C22—H221	120.6
C8—C9—C10	119.7 (4)	P4—C23—C24	118.0 (3)
C8—C9—H91	120.1	P4—C23—C28	122.1 (3)
C10—C9—H91	120.2	C24—C23—C28	119.8 (3)
C9—C10—C5	120.1 (4)	C23—C24—C25	119.3 (4)
C9—C10—H101	119.9	C23—C24—H241	120.3
C5—C10—H101	120.0	C25—C24—H241	120.3
P4—C11—C12	119.1 (3)	C24—C25—C26	120.3 (4)
P4—C11—C16	121.3 (3)	C24—C25—H251	119.8
C12—C11—C16	119.5 (4)	C26—C25—H251	119.9
C11—C12—C13	121.0 (5)	C25—C26—C27	119.9 (4)
C11—C12—H121	119.6	C25—C26—H261	120.0
C13—C12—H121	119.5	C27—C26—H261	120.0
C12—C13—C14	119.4 (5)	C26—C27—C28	120.5 (4)
C12—C13—H131	120.3	C26—C27—H271	119.7
C14—C13—H131	120.3	C28—C27—H271	119.8
C13—C14—C15	121.2 (4)	C23—C28—C27	120.1 (3)
C13—C14—H141	119.4	C23—C28—H281	120.0
C15—C14—H141	119.4	C27—C28—H281	119.9

Symmetry codes: (i) −*x*, *y*, −*z*+3/2.

## References

[bb1] Altomare, A., Cascarano, G., Giacovazzo, G., Guagliardi, A., Burla, M. C., Polidori, G. & Camalli, M. (1994). *J. Appl. Cryst.* **27**, 435.

[bb2] Betteridge, P. W., Carruthers, J. R., Cooper, R. I., Prout, K. & Watkin, D. J. (2003). *J. Appl. Cryst.* **36**, 1487.

[bb3] Bruker (2005). *APEX2* Version 1.27. Bruker AXS Inc., Madison, Wisconsin, USA.

[bb4] Dance, I. G. & Scudder, M. L. (1995). *J. Chem. Soc. Chem. Commun.* pp. 1039–1040.

[bb5] Dance, I. G. & Scudder, M. L. (1996). *J. Chem. Soc. Dalton Trans.* pp. 3755–3769.

[bb6] Dowty, E. (2000). *ATOMS* Version 6.1. Shape Software, Hidden Valley Road, Kingsport, Tennessee, USA.

[bb7] Hasselgren, D., Dean, P. A. W., Scudder, M. L., Craig, D. C. & Dance, I. G. (1997). *J. Chem. Soc. Dalton Trans.* pp. 2019–2027.

[bb8] Müller, A., Diemann, E., Jostes, R. & Bögge, H. (1981). *Angew. Chem. Int. Ed. Engl.* **20**, 934–955.

[bb9] O’Neal, S. C. & Kolis, J. W. (1988). *J. Am. Chem. Soc.* **110**, 1971–1973.

[bb10] Parvez, M., Boorman, P. M. & Wang, M. (1997). *Acta Cryst.* C**53**, 413–414.

[bb11] Ruhlandt-Senge, K. & Müller, U. (1990). *Z. Naturforsch. Teil B*, **45**, 995–999.

[bb12] Sasvári, K. (1963). *Acta Cryst.* **16**, 719–724.

[bb13] Sheldrick, G. M. (1996). *SADABS* University of Göttingen, Germany.

